# Evidence of Hermaphroditism and Sex Ratio Distortion in the Fungal Feeding Nematode *Bursaphelenchus okinawaensis*

**DOI:** 10.1534/g3.114.012385

**Published:** 2014-08-12

**Authors:** Ryoji Shinya, Koichi Hasegawa, Anthony Chen, Natsumi Kanzaki, Paul W. Sternberg

**Affiliations:** * Howard Hughes Medical Institute and Division of Biology and Biological Engineering, California Institute of Technology, Pasadena, California 91125; † Department of the Environmental Biology, College of Bioscience and Biotechnology, Chubu University, Kasugai 487-8501 Japan; ‡ Forestry and Forest Products Research Institute, Tsukuba 305-8687, Japan

**Keywords:** hermaphroditism, nematode, early embryogenesis, genetics, satellite model

## Abstract

Nematodes have many different reproductive strategies along with their divergent life histories; the ability of hermaphrodite to self- and cross-fertilize is useful for genetic manipulation. Here, we demonstrate the hermaphroditism of the fungal feeding nematode *Bursaphelenchus okinawaensis*, which was formerly described as a parthenogenetic nematode, and we show its other unique sexual characteristics. To determine that it is hermaphroditic, we performed the following experiments: observation of the pronuclear and chromosome behavior during oogenesis and early embryogenesis; observation of spermatogenesis during the fourth larval stage; investigation of sperm utilization; and investigation of phenotypic segregation after cross-mating using a chemically induced visible mutant. We then investigated the mating preferences and spermatid size difference between males and hermaphrodites. *B. okinawaensis* males successfully mated only with sperm-depleted old hermaphrodites, and the spermatid sizes of males were almost the same as those of hermaphrodites. Moreover, the sex ratio of cross-fertilized progeny was highly skewed toward hermaphrodites. *B. okinawaensis* is phylogenetically distant from established model nematodes such as *C. elegans* and is more closely related to some economically relevant parasitic nematodes. This newly discovered hermaphroditic nematode has great potential for evolutionary and parasitological research.

Since Sydney Brenner described the genetics of *Caenorhabditis elegans* (Brenner 1974), it has been extensively studied as a model organism. For the past 40 years, a wide variety of techniques, tools, and information has accumulated, and these achievements have continuously increased the value of *C. elegans* study (Wood 1988; Riddle *et al.* 1997; Girard *et al.* 2007; Antoshechkin and Sternberg 2007). Brenner focused on *C. elegans* because of its short generation time, ease of cultivation, and an unusual reproductive mode in which the presence of self-fertilizing hermaphrodites that can nonetheless mate with males for cross-fertilization is very useful for genetic studies. In addition, its small transparent body and simple nervous system were also superb for studying development and the nervous system (Brenner 2009). These biological characteristics have allowed powerful genetic screening in *C. elegans*.

Because nematodes comprise one of the most diverse animal groups, they have a strong potential for evolutionary studies (Fitch 2005). In addition, the genetic tools and techniques developed in *C. elegans* seem to be useful in understanding animal and plant parasitic diseases. However, the number of species that are useful for such sophisticated genetics is limited (Sommer and Streit 2011). Only three other nematode genetic systems, using the free-living bacteriovores *C. briggsae* (Gupta *et al.* 2007: Koboldt *et al.* 2010), *Pristionchus pacificus* (Sommer *et al.* 1996; Sommer 2006), and *Oscheius tipulae* (Felix 2006), have been established and used for comparative biological studies. These genetic systems are excellent models for researching the evolution of development but are not well-suited for studying parasitism because all of these are phylogenetically distant from most economically relevant parasites (Dieterich and Sommer 2009). There are two major obstacles to performing genetic studies in nematodes, especially parasitic nematodes, compared with the free-living bacteriovores discussed above. First, most nematodes are gonochoristic (male/female). Second, most parasites are difficult to culture on artificial media.

One genus that might be well-suited for the laboratory study of plant parasites is *Bursaphelenchus* (Hasegawa and Miwa 2008; Dieterich and Sommer 2009), which consists of more than 100 species and contains two economically relevant plant pathogens, namely *B. xylophilus* and *B. cocophilus*, as well as a few other potential plant pathogens (Kanzaki 2008). *Bursaphelenchus* nematodes feed on fungi and/or plant cells using their stylet and have three well-developed esophageal gland cells, as do other major plant parasitic nematodes. The esophageal gland cells of plant parasitic nematodes produce many secreted effector proteins and have essential roles in their plant parasitism (Hussey *et al.* 2002; Shinya *et al.* 2013). Furthermore, most *Bursaphelenchus* species have a phoretic relationship with one or more types of insects (*e.g.*, longhorn beetles, bark beetles, weevils, bees). Phylogenetically, *Bursaphelenchus* is assigned to Clade IV, along with other major plant parasitic nematodes (*e.g.*, *Meloidogyne*, *Heterodera*) by Blaxter *et al.* (1998), although *Caenorhabditis*, *Pristionchus*, and *Oscheius* are members of nematode Clade V. *Bursaphelenchus* can easily be cultured on artificial media and have a short life cycle compared with other parasitic nematodes. However, their gonochoristic reproductive mode has prevented efficient genetic studies (Shinya *et al.* 2012).

In 2008, Kanzaki *et al.* (2008) reported the discovery of an apparently parthenogenetic *Bursaphelenchus* species, *Bursaphelenchus okinawaensis*. In their study, they suggested that the reproductive mode of *B. okinawaensis* is parthenogenesis based on the following evidence: no hermaphroditism had been reported in the superfamily *Aphelenchoidea*; most females had empty spermathecae, and they could not confirm the presence of developing sperm; the sperm deposited in the spermathecae of females were almost the same size as those in males, although male sperm are larger than hermaphrodite sperm in *C. elegans* and several other hermaphroditic species; and no ovotestes were observed in young females. In this study, we first observed early embryogenesis and spermatogenesis to determine the precise reproductive mode of *B. okinawaensis*; to determine the reproductive mode as parthenogenesis or hermaphroditism, the observation of pronuclear fusion in the early embryo and chromosome behavior is crucial. After determining that *B. okinawaensis* is indeed hermaphroditic, we wanted to demonstrate outcrossing by transmission genetics. We thus performed ethyl methanesulfonate (EMS) mutagenesis, isolated mutations, and used them to demonstrate transmission through males and Mendelian segregation in progeny hermaphrodites.

## Materials and Methods

### Nematode isolation

Three strains of *Bursaphelenchus okinawaensis* (NK212, SH1, SH2) were used in this study. NK212 was isolated in 2005 from an adult female of the beetle *Monochamus maruokai* and was used in a previous descriptive taxonomic article (Kanzaki *et al.* 2008). In 2012, we isolated two new strains, SH1 and SH2, from the tracheal system of an adult male and an adult female, respectively, of *M. maruokai*. All of the *M. maruokai* were caught on Ishigaki Island, Okinawa, Japan. All of these strains are apparently indistinguishable from each other.

### Culture and maintenance

In all experiments except for the egg collecting procedure, the nematode *B. okinawaensis* was propagated on the budding yeast *Saccharomyces cerevisiae*, strain W303-1A, on 1/10 malt extract agar medium (MEA) (Difco) with 4% agar containing 100 µg/mL chloramphenicol in a 60-mm Petri dish (hereafter called 1/10 MEA-chloramphenicol plate). For the egg collection procedure, the nematode was propagated on the filamentous fungus *Botrytis cinerea* on MEA plates with 4% agar containing 100 µg/ml chloramphenicol in a 90-mm Petri dish.

### Observation of pronuclear fusion in early embryo of *B. okinawaensis*

For light microscope observation, gravid worms propagated on yeast culture plates were picked up using a fine needle and transferred onto a 2% agarose pad prepared on a glass microscope slide. The worms were incubated for 5 to 10 min to be allowed to lay eggs on the agarose pad. After they laid eggs, all of the worms were removed and the agarose pad was covered with a cover glass and sealed with silicon grease. The eggs laid were observed until the four-cell stage under differential interference contrast (DIC) optics. This experiment was performed with 10 individual biological replicates. For observation of chromosome behavior during oogenesis and embryogenesis, gravid worms propagated on 1/10 MEA-chloramphenicol plates were picked up and transferred into a droplet of 0.1 M NaCl in the well of an eight-well glass slide (Hasegawa *et al.* 2004). The worms were incubated for 5 to 50 min to allow egg laying in the well. The 0.1 M NaCl was completely exchanged for −20° methanol by pipetting, incubated for 5 min, and then stained with 2 µg/mL of DAPI in phosphate-buffered saline (PBS) for 10 min. After washing twice with PBS, the worms were mounted in Vectashield (Vector Laboratories Inc., USA). The DAPI-stained images were obtained with a confocal laser-scanning microscope (LSM5 exciter; Carl Zeiss).

### Observation of spermatogenesis and oogenesis in hermaphrodites

To observe the process of spermatogenesis and oogenesis, the development of nematodes was synchronized by allowing the second larval stage (L2) to hatch in the absence of food (Shinya *et al.* 2009). The arrested L2 worms were collected and transferred onto the edge of a lawn of *S. cerevisiae* on 1/10 MEA-chloramphenicol plates. Worms were collected every 6 hr and mounted onto 2% agarose pads with a drop of M9 buffer containing 10 mM sodium azide solution as an anesthetic to observe the development of the gonads under Nomarski DIC optics. Observation was continued until no more eggs were laid.

### The relationship between the number of the progeny and the number of sperm in spermatheca in hermaphrodites

To count the number of progeny, a single L4 stage hermaphrodite was picked and transferred to a 1/10 MEA-chloramphenicol plate with yeast lawn. The hermaphrodite was transferred to a fresh plate every day until no more eggs were laid, and the number of progeny was counted every day. This experiment was performed in eight biological replicates.

The number of sperm cells in the spermatheca was measured using DAPI staining. Late L4 hermaphrodites (48–52 hr after hatching at 25°) were collected and used for the sperm counting experiment. Hermaphrodites were picked up and transferred into a droplet of 0.1 M NaCl in the well of a poly-L-lysine–coated eight-well glass slide. The fixation and staining were performed using the aforementioned protocol. The number of sperm cells was counted under a confocal laser-scanning microscope (LSM5 exciter; Carl Zeiss).

### Isolation and characterization of mutants

Mutations were generated by treatment with EMS as described by Brenner (1974), with some modifications. Young adult hermaphrodites of the SH1 strain were collected and washed three times with sterilized ddH_2_O. The nematodes were suspended and incubated in 75 to 100 µM EMS in M9 buffer for 4 hr at room temperature. Nematodes were then washed three times with M9 buffer and transferred to the edge of the 1/10 MEA-chloramphenicol plate with yeast lawn. After overnight incubation at 25°, three to five mutagenized worms were transferred onto a new yeast plate and allowed to lay eggs for 1 to 2 d (approximately 30 F_1_ eggs). After 7 to 14 d at 25°, the F_2_ generation was screened for visible makers. Approximately 90,000 gametes were screened after EMS mutagenesis. Although more than 400 candidate mutants were originally picked, approximately 90% of the candidates picked turned out to be sterile or had a variable phenotype and were discarded.

All mutants of *B. okinawaensis* were stored using the soft agar freezing method developed for *C. elegans* (Stiernagle 2006) with small modifications. The nematodes were each grown on a 90-mm MEA-chloramphenicol plate with yeast lawn. After 1 to 3 wk of incubation at 25°, the worms were washed off with M9 buffer from plates that had a large number of L2-L3 stage worms. The worms were collected in 15-mL centrifugation tubes and washed five times with M9 buffer. An equal volume of molten soft agar freezing solution (1.74 g NaCl, 2.04 g KH_2_PO_4_, 1.68 mL of 1 M NaOH, 1.2 g agar, 90 g glycerol, ddH_2_O to 300 mL, autoclave) was added into the tubes and mixed well. The worms were transferred to 1.8-mL cryovials (330 µL of worm suspension each), allowed to equilibrate to −80° insulated in a small Styrofoam box, and stored in cardboard boxes at −80°.

### Male mating ability

Male mating ability was investigated using the *Bok-rol*(*sy762)* mutant strain because this mutant is fertile and has a clear roller phenotype. Mating plates were prepared by placing five wild-type males of mixed ages along with a single adult *Bok-rol*(*sy762)* hermaphrodite (aged either 1 d, 3 d, or 6 d after the final molt) on 1/10 MEA-chloramphenicol plates with yeast lawns. These plates were incubated at 25°; 24 hr later, the hermaphrodites were transferred to new plates every following day and allowed to lay eggs until they stopped laying eggs. Eggs were allowed to hatch and progeny were grown until phenotype and sex could be determined.

### Sperm size measurement

Young adult hermaphrodites and young males were collected from synchronized yeast culture plates. Worms were placed in a drop of sperm medium buffer (5 mM HEPES sodium salt pH 7.4, 50 mM NaCl, 25 mM KCl, 5 mM CaCl_2_, 1 mM MgSO_4_, 10 mM dextrose) and dissected to isolate sperm (Nelson and Ward 1980); they were then observed by DIC microscopy. The cross-sectional area of spherical spermatids was measured using ImageJ software. A total of 100 sperm from 80 hermaphrodites and 126 sperm from 24 males were measured.

### Classification of mutants

A total of 33 fertile mutants with stable visible phenotypes were crossed with wild-type males and classified. Brenner (1974) sorted his *C. elegans* mutants into five classes: autosomal recessive; sex-linked recessive; autosomal semidominant; sex-linked semidominant; and dominant. However, we did not distinguish between autosomal and sex-linked characters because our mating experiment in *B. okinawaensis* showed that the frequency of male progeny after crosses was low. The mating plates were prepared by placing five wild-type (WT) males along with two sperm-depleted 6-d-old adult hermaphrodites of each mutant on 1/10 MEA-chloramphenicol plates with yeast lawns. The plates were incubated at 25° for 24 hr, and then the hermaphrodites that were used for the mating were transferred to new plates and allowed to lay eggs for 48 hr. The old adult hermaphrodites were then removed and progeny were grown until phenotypes could be determined. Three to ten replicates were examined for each mutant.

## Results

### Pronuclear fusion in early embryogenesis

**Figure 1:**
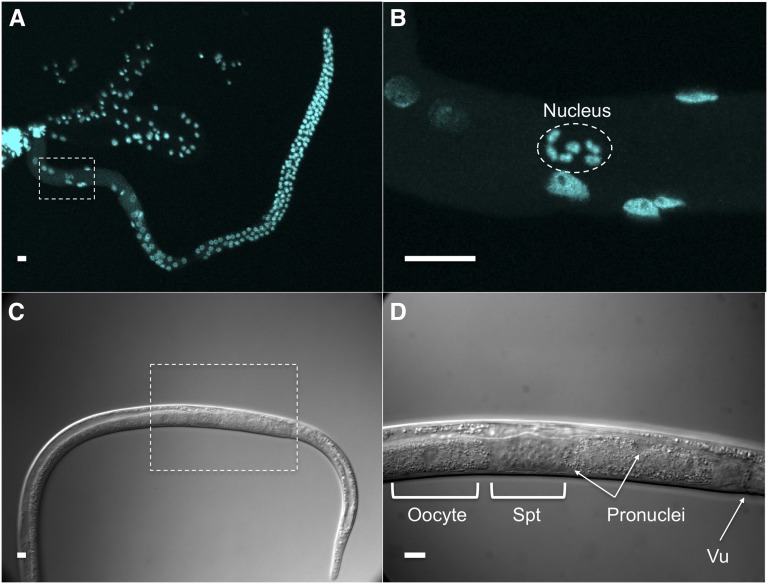
The germ line of *Bursaphelenchus okinawaensis*. (A, B) Confocal images showing DAPI staining of a gonad dissected from an adult hermaphrodite. The oocyte stored in the proximal gonad is boxed in (A) and shown at higher magnification in (B). (C and D) Differential interference contrast images of the reproductive system of a gravid adult hermaphrodite. The egg in the uterus is boxed in (C) and shown at higher magnification in (D). Vu, vulva; Spt, spermatheca. Scale bar represents 10 μm.

**Figure 2:**
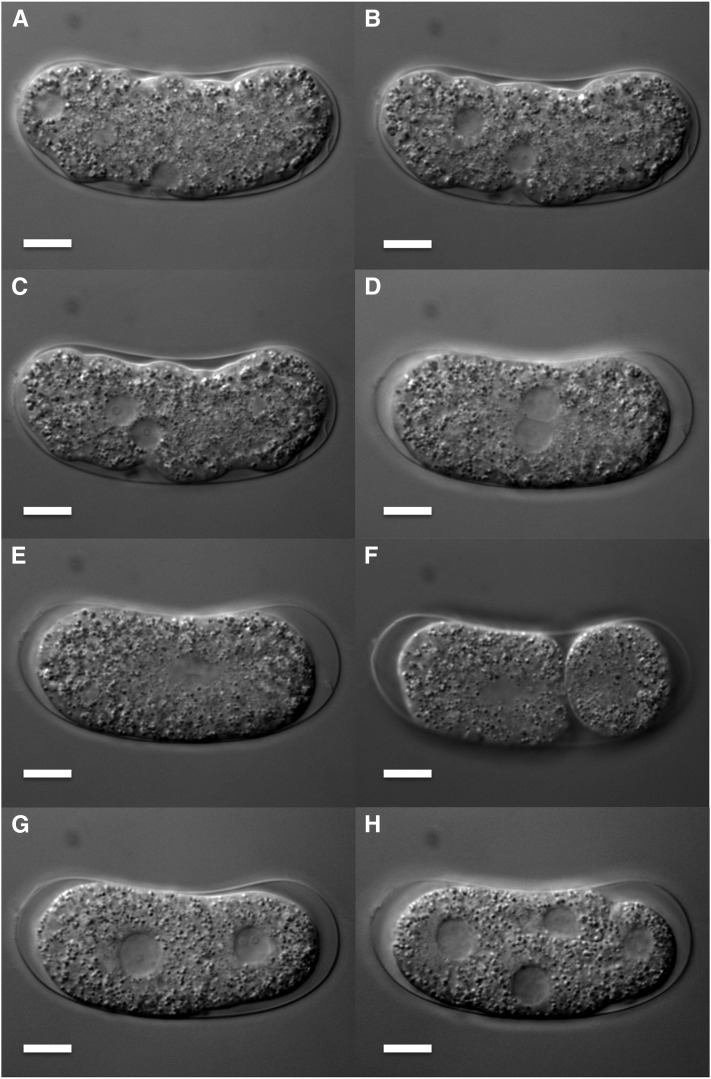
Early embryogenesis of *Bursaphelenchus okinawaensis* through four-cell stage. (A–E) Pronuclear migration and fusion. (F) Asymmetric first cleavage. (G) Two-cell stage. (H) Four-cell stage. Scale bar represents 10 μm.

**Figure 3:**
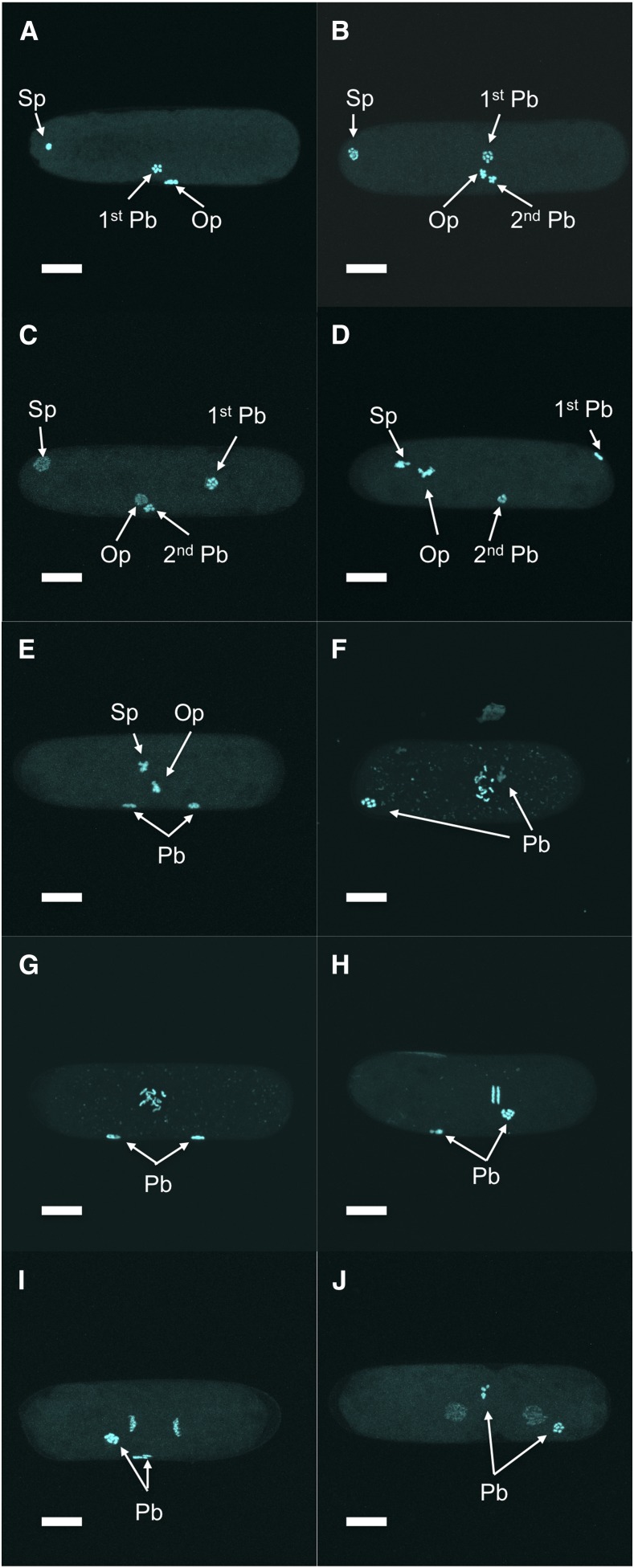
Chromosome behavior and polar body extrusion of *Bursaphelenchus okinawaensis* during early embryogenesis. (A) First polar body extrusion. (B) Second polar body extrusion. (C) Emergence of two pronuclei. (D–F) Pronuclear migration. (G) Pronuclear fusion. (H–J) First cleavage. Sp, sperm pronucleus; Op, oocyte pronucleus; Pb, polar body. Scale bar represents 10 μm.

Oocytes stored in the proximal gonad of *Bursaphelenchus okinawaensis* each had a single germinal vesicle containing six sister chromatids (Figures 1, A and B). After the oocyte moved through the spermatheca to the uterus, the two pronuclei were reconstructed (Figures 1, C and D) and the fertilized egg was laid soon thereafter (after approximately 20 min). At this time, one pronucleus appeared at one pole of the embryo and the other pronucleus emerged at a lateral position (Figure 2A). The first and second polar bodies were extruded from the pronucleus at a lateral position (Figures 3, A and B). This result suggested that the pronucleus at the lateral position was oocyte-derived while the other pronucleus was sperm-derived. After the oocyte pronucleus completed meiosis, the two pronuclei migrated toward each other (Figure 2B and Figure 3, C and D). After the pronuclei met, they moved to the center of the embryo and rotated 30° to 90° (Figure 2, C and D and Figure 3, E and F) and then fused (Figure 2E and Figure 3G). At this time, the number of chromosomes was restored from six to 12 (Figure 3G). Subsequently, first and second cleavages occurred (Figures 2, F–H and Figures 3, H–J). No significant differences in the process of pronuclear fusion were observed between three *B. okinawaensis* isolates (only the images from SH1 are shown in Figure 1).

### Spermatogenesis and oogenesis in the hermaphrodite

**Figure 4:**
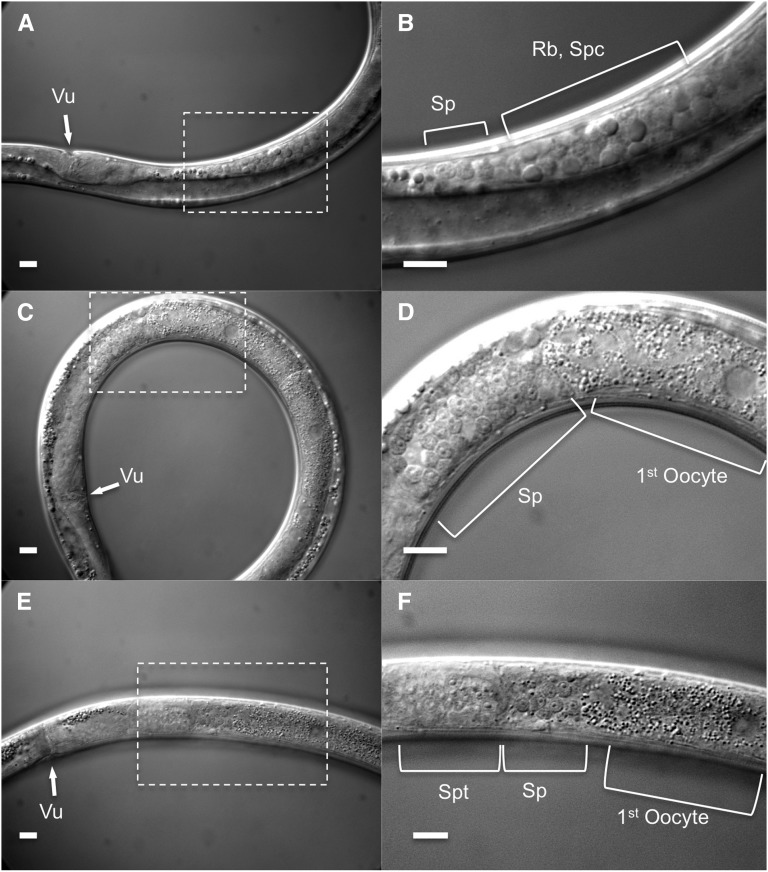
Spermatogenesis in L4 hermaphrodite of *Bursaphelenchus okinawaensis*. (A and B) Spermatogenesis in the proximal gonad. (C and D) Transition from spermatogenesis to oogenesis. (E and F) The spermatids are pushed into the spermatheca by the first oocyte during the first ovulation. Vu, vulva; Sp, spermatid; Rb, residual body; Spc, spermatocyte; Spt, spermatheca. Scale bar represents 10 μm.

Sperm development was observed in hermaphrodites using Nomarski DIC microscopy. Spermatogenesis occurred in the proximal part of the gonad (Figure 4A) during the early L4 stage. At the time when spermatogenesis started, no oocytes were observed in the gonad (Figure 4, A and B); oocytes were only generated after spermatogenesis had ceased (Figure 4, C and D). After the animals had produced a few oocytes, the clear structure of the spermatheca was first observed and the spermatids moved into the spermatheca (Figure 4, E and F). Spermatogenesis occurred only once in the lifespan.

### The numbers of self-produced sperm and of progeny

**Figure 5:**
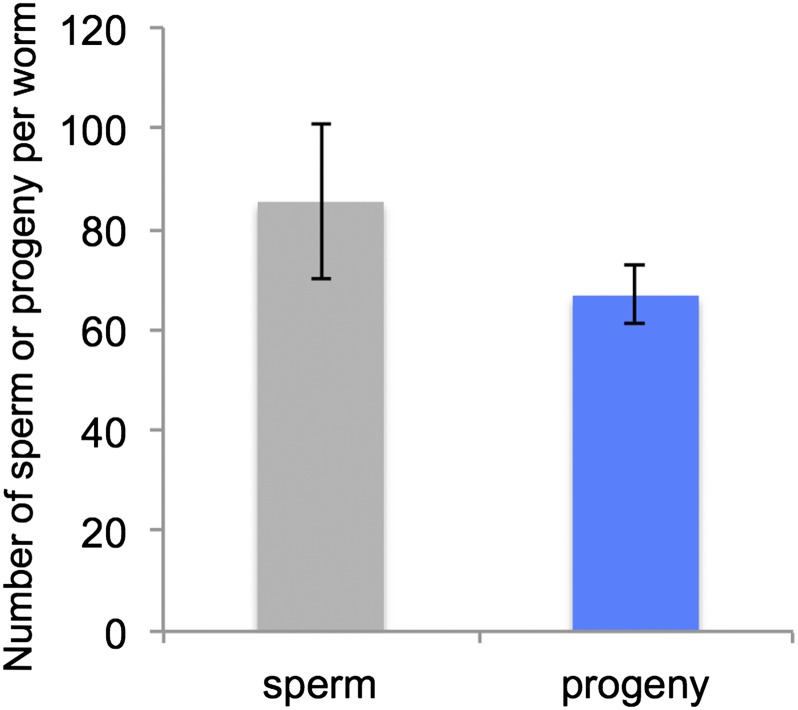
The number of self-produced sperm and progeny in *Bursaphelenchus okinawaensis*. The numbers presented are mean values of eight biological replicates. Error bars represent SD.

To investigate the utilization of self-produced sperm, we counted the number of self-produced sperm stored in unmated young hermaphrodites and the number of self-progeny. All of the young adult hermaphrodites had sperm in the spermatheca; the average number of sperm was 85 ± 15.5 per worm (mean ± SD, N = 8) (Figure 5). Hermaphrodites laid eggs for 4 to 5 d and the total number of progeny was 67 ± 5.8 per worm (mean ± SD, N = 8) (Figure 5). After hermaphrodites stopped laying eggs, no sperm were observed in either spermatheca.

### Isolation and characterization of mutants

We performed EMS mutagenesis to isolate visible genetic markers in *B. okinawaensis*. We screened 1500 plates each with 30 F_1_ progeny per plate (approximately 90,000 gametes) and more than 400 candidate mutants were initially isolated. Most of these candidates were sterile or had a variable phenotype and were discarded. Ultimately, 33 mutants were kept and frozen in glycerol stocks (Table 1). These comprise 11 Dumpy (Dpy), 12 Roller (Rol), and 10 Uncoordinated (Unc) mutants.

**Table 1:** List of mutants isolated by EMS mutagenesis

Phenotype	Allele	Comments
Dpy	*sy751*	Medium dumpy
	*sy752*	Medium dumpy, slightly sluggish
	*sy753*	Weak dumpy
	*sy754*	Weak dumpy, slightly sluggish, coiler
	*sy755*	Medium dumpy, sluggish
	*sy756*	Medium dumpy, right-handed roller
	*sy757*	Weak dumpy, right-handed roller
	*sy758*	Variable dumpy, sluggish, coiler
	*sy759*	Weak dumpy
	*sy760*	Strong dumpy, slightly sluggish
	*sy761*	Medium dumpy
Rol	*sy762*	Right-handed roller (L2 wild-type)
	*sy763*	Right-handed roller (L2 wild-type)
	*sy764*	Right-handed roller (L2 wild-type)
	*sy765*	Right-handed roller (L2 wild-type)
	*sy766*	Left-handed roller (L2 weak roller), Slightly dumpy
	*sy767*	Variable right-handed roller (L2 wild-type)
	*sy768*	Left-handed roller (L2 wild-type), slightly small
	*sy769*	Right-handed roller (L2, L3 wild-type)
	*sy770*	Left-handed roller (L2 wild-type), small
	*sy771*	Left-handed roller (L2 weak roller), small
	*sy772*	Left-handed roller (L2 wild-type), small
	*sy773*	Left-handed roller (L2, L3 variable phenotype)
Unc	*sy774*	Sluggish, weak dumpy, coiler
	*sy775*	Kinker, sluggish, slightly small
	*sy776*	Kinker, sluggish
	*sy777*	Strong sluggish (almost paralyzed), Small, coiler
	*sy778*	Kinker, sluggish, slightly small
	*sy779*	Slightly sluggish
	*sy780*	Small, coiler
	*sy781*	Sluggish
	*sy782*	Weak kinker
	*sy783*	Weak kinker

#### Dumpy mutants:

**Figure 6:**
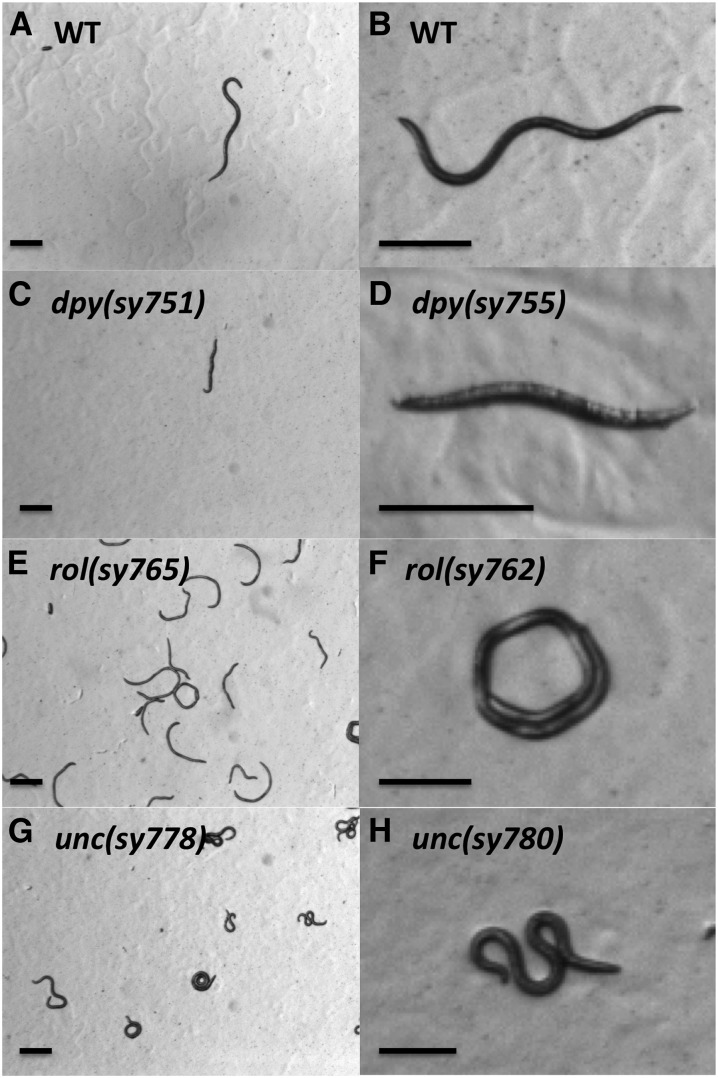
Stereomicroscope images of wild-type and mutant strains of *Bursaphelenchus okinawaensis*. (A and B) WT, wild-type. (C and D) Dpy, Dumpy. (E and F) Rol, Roller. (G and H) Unc, Uncoordinated. Scale bar represents 200 μm.

The body shape of the *B. okinawaensis* wild-type is significantly narrower than that of *C. elegans* (Figure 6A). Eleven Dpy mutants that were shorter than the wild-type were isolated (Figure 6B). The frequency of appearance of candidate Dpy mutants was relatively high, but most of these Dpy animals were sterile. Some of the Dpy mutants were also roller and slightly sluggish.

#### Roller mutants:

Twelve Rol mutants with helically twisted bodies were isolated. The Rol mutants were only able to move in a circle rather than a wavy line (Figure 6C), as does the wild-type. We isolated both right-handed and left-handed rollers, as previously observed among *C. elegan* Rol mutants. In most of the Rol mutants, the phenotype is clearly expressed and recognizable only in L4 and adult stages. Such a stage-specific expression of Rol phenotypes was also observed in *C. elegans* (Brenner 1974; Cox *et al.* 1980).

#### Uncoordinated mutants:

Ten Unc mutants that exhibited abnormal locomotion were isolated. Although wild-type *B. okinawaensis* display smooth locomotion on an agar plate, these Unc mutants showed sluggish, kinker, coiler, or paralyzed locomotion (Figure 6D). In *C. elegans*, many Unc mutants have been isolated. It is more difficult to recognize the abnormal locomotion in *B. okinawaensis* than in *C. elegans*. This could be because the movement of *B. okinawaensis* is normally slower than that of *C. elegans* and because *B. okinawaensis* animals tend to stay on the yeast lawn, which is not transparent.

### Mating experiments

To distinguish between progeny resulting from self- or cross-fertilization, we used adult *Bok-rol*(*sy762)* mutant hermaphrodites (1 d, 3 d, and 6 d after the final molt). The Rol phenotype of the *Bok-rol*(*sy762)* strain is recessive. Therefore, the F_1_ Rol progeny result from self-fertilization, and the non-Rol progeny result from cross-fertilization. In day 1 and day 3 adult hermaphrodites, which possess self-produced sperm, all of the F_1_ progeny had a roller phenotype (Table 2). During the experiment, *B. okinawaensis* males were not chemotactically attracted to day 1 or day 3 adult hermaphrodites. In the absence of males, day 6 adult hermaphrodites would have exhausted their sperm and stopped laying eggs; however, they produced wild-type (cross) progeny when mated (Table 2). This result suggested that *B. okinawaensis* males could not mate with hermaphrodites that still had self-produced sperm and were still laying eggs. Males did not attempt to mate within the first 5 min with young hermaphrodites that still possessed self-produced sperm. We also observed the phenotypes of F_2_ progeny of self-fertilization in the F_1_ cross-progeny from this cross. The phenotypic ratio of F_2_ progeny was almost exactly 3:1 (336 WT and 120 Rol).

**Table 2:** Mating tests using different ages of hermaphrodites

Type of Mother*^a^*	N	Self-Sperm Status*^b^*	F_1_ Phenotype		% Male F_1_ Progeny	F_2_ Phenotype After Self-Fertilization	
			Rol (Self)	WT (Cross)		Rol	WT
Rol day 1 adult	16	+	971	0	0.5	489	0
Rol day 3 adult	16	+	318	0	0.6	512	0
Rol day 6 adult	30	−	0	909	1.8	120	336
WT day 6 adult	12	−	0	635	0.3	N/A	N/A

Rol animals were homozygous for *Bok-rol*(*sy762)*. WT, wild-type; N/A, not applicable; N, number of hermaphrodites used.

a Day 1 adult, day 3 adult, and day 6 adult indicate that the adult hermaphrodite had been incubated for 1, 3, or 6 d after final molt in the absence of males, respectively.

b Day 1 adult and day 3 adult hermaphrodites possessed self-produced sperm in their spermatheca or uteri at the start of the mating experiment. Day 6 adult hermaphrodites had exhausted self-produced sperm and stopped laying eggs by the start of the mating experiment.

The sex ratio of cross-fertilized F_1_ progeny was highly skewed (893 hermaphrodites and 16 males) (Table 2). However, the male ratio of the self-fertilized progeny of *Bok-rol*(*sy762)* mutant was 0.5%. To eliminate the possibility that the mutation induced such a skewed sex in cross-fertilized progeny, we performed a mating experiment using the sperm-depleted wild-type hermaphrodite (SH1 strain) with the wild-type male. After mating, the sex ratio of the resulting cross-progeny was 99.7% hermaphrodite (Table 2). We also performed mating experiments in NK212 and SH2 strains and observed a strong hermaphrodite-biased sex ratio (data not shown). These results demonstrate that the hermaphrodite bias among progeny does not result from the *Bok-rol*(*sy762)* mutation. Moreover, the sex ratio bias is not a strain-specific trait.

### Sperm size

**Figure 7:**
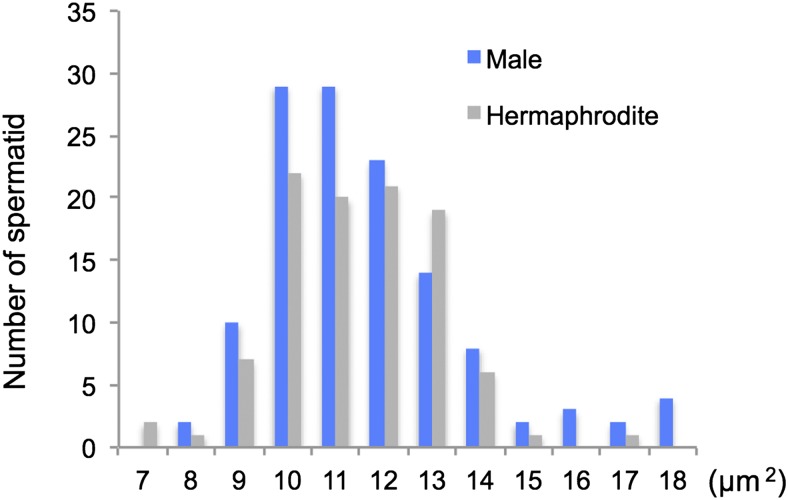
Distribution of spermatid cross-sectional areas in hermaphrodites and males in *Bursaphelenchus okinawaensis*. The cross-sectional area of spherical spermatids was measured using ImageJ software. A total of 100 sperm from 80 hermaphrodites and 126 sperm from 24 males were measured.

The average sperm size, as measured by the cross-sectional area, of males and hermaphrodites of *B. okinawaensis* was 12.2 μm^2^ and 11.9 μm^2^, respectively. There is no significant difference in the distribution of spermatid size between males and hermaphrodites (*P* = 0.61, one-way ANOVA) (Figure 7).

### Classification of mutants

All the crosses between wild-type males and mutant hermaphrodites produced F_1_ progeny with a wild-type phenotype, indicating that all 33 mutant phenotypes are recessive. During these experiments, no F_1_ cross-progeny male with a mutant phenotype was observed, although the number of males was low (0–2 males per plate) and in a few strains no males were observed.

## Discussion

We have described the hermaphroditism of *B. okinawaensis*, which was formerly described as a parthenogenetic nematode. We also show its other unique sexual characteristics. This is the first report of a hermaphroditic species in the superfamily Aphelenchoidea and in fungal feeding nematodes. Furthermore, our work establishes EMS mutagenesis, freezing, and mating methods that allow genetic studies in this species. Thus, we demonstrate that this close relative (sister species of the "*xylophilus*" group) of *B. xylophilus*, the pathogen causing pine wilt disease, is amenable to genetic analysis.

### Polar body extrusion and pronuclear fusion

In parthenogenetic organisms, a female gamete develops a new individual without being fertilized by a male gamete (sperm). The mechanisms of parthenogenesis have been divided into two main types: apomictic and automictic (Pearcy *et al.* 2011). In apomictic parthenogenesis, the embryo develops in the absence of a completed meiosis or of gamete fusion, and the offspring are true clones of the mother. In automictic parthenogenesis, meiosis occurs and diploidy is restored by fusion of two nuclei originating from the same primary oocyte or by gamete duplication. In the case of *B. okinawaensis* embryogenesis (Figure 1, Figure 2, Figure 3), one of the pronuclei appeared at the pole of the embryo before completion of the first meiosis of the oocyte-derived pronucleus. Moreover, the extruded polar bodies were not fused with the oocyte-derived pronucleus through embryogenesis. The pronuclear and polar body behaviors in *B. okinawaensis* were inconsistent with those of parthenogenesis and essentially the same as in the gonochoristic species *B. xylophilus* (Hasegawa *et al.* 2004). This is conclusive proof of their hermaphroditism.

### Spermatogenesis and self-produced sperm utilization

We also observed spermatogenesis and oogenesis in the L4 stage, and we investigated the number of sperm and progeny. The *B. okinawaensis* hermaphrodites have a single-armed (monodelphic) gonad, which extends straight forward from the vulva (proximal region) toward the anterior (distal region). Spermatogenesis was clearly observed within the proximal gonad in the early L4 stage prior to oogenesis (Figure 4). Once formed, spermatids are pushed into the spermatheca by the first oocyte during the first ovulation. This spermatogenesis process is essentially the same as in *C. elegans* (Ward and Carrel 1979). Although Kanzaki *et al.* (2008) reported that most females had empty spermathecae, and they could not confirm developing sperm, we observed many sperm in all hermaphrodites we examined, regardless of the *B. okinawaensis* strain. In the previous report, they used a limited number of fixed specimens for observation of spermatogenesis. This could be the cause of the difference between our results and the previous report (Kanzaki *et al.* 2008). Our DAPI staining experiments showed that young adult hermaphrodites of *B. okinawaensis* stored an average of 85 sperm cells in the spermatheca. Sperm utilization experiments showed that unmated hermaphrodites of *B. okinawaensis* laid eggs for 4 to 5 d and produced an average of 67 self-progeny per worm (Figure 5). Furthermore, no sperm or only a few sperm were observed in the spermatheca or uterus after hermaphrodites stopped laying eggs. This result indicates that sperm are utilized for producing progeny. The utilization of self-produced sperm also supports their hermaphroditic reproduction. Similar differences between the numbers of sperm and self-progeny have been reported in unmated *C. elegans* hermaphrodites (Nigon 1949; Ward and Carrel 1979). Specifically, Nigon (1949) reported that hermaphrodites of the Bergerac strain of *C. elegans* produced 264 progeny and 370 sperm per worm. Also, Ward and Carrel (1979) reported that hermaphrodites of the N2 strain in *C. elegans* produced 234 progeny and 253 sperm per worm. Some unused sperm may have been pushed out of the body through the vulva during egg laying; others may have been defective and incapable of productively fertilizing an oocyte.

### Phenotypic segregation

The results of phenotypic segregation experiments are most consistent with hermaphroditism but do not fully rule out automictic parthenogenesis. After cross-mating between wild-type males and Rol hermaphrodites that had exhausted their endogenous sperm, all of the F_1_ progeny showed a wild-type phenotype (non-Rol). In the F_2_ self-progeny produced by these animals, the phenotype ratio of non-Rol and Rol in the F_2_ generation was almost exactly 3:1, which corresponds to segregation of a single Mendelian locus. It is known that chromosomes of *C. elegans* are holocentric and only one cross-over per chromosome pair per meiosis occurs (Hillers and Villeneuve 2003). From the shape of the segregating chromosomes at anaphase (Figure 3, H and I), the chromosomes of *B. okinawaensis* would be holocentric or polycentric rather than monocentric. If chromosomes of *B. okinawaensis* are holocentric and *Bok-rol*(*sy762)* is unlinked to the pairing center at the far end of its chromosome, then automictic parthenogenesis might give a 3:1 phenotype ratio in the F_2_ generation. Therefore, the results of the phenotypic segregation experiments do not rule out the possibility of automictic parthenogenesis. However, automictic parthenogenesis gives many different phenotype ratios, and thus most versions are ruled out by our data.

### EMS mutagenesis and frozen glycerol stocks

We recovered 33 visible, recessive mutants of *B. okinawaensis* after EMS mutagenesis (Table 1). Because we screened approximately 90,000 gametes, the apparent efficiency of visible mutant isolation was lower than the frequencies in *C. elegans* (Brenner 1974), *P. pacificus* (Sommer *et al.* 1996), and *O. tipulae* (Felix 2006). The low recovery of mutations in *B. okinawaensis* may be due to resistance to mutation or to uptake of EMS. Further optimization of EMS mutagenesis protocol in *B. okinawaensis* might produce a higher frequency. The availability of existing mutations will enable more accurate assessments of mutation frequency such as noncomplementation screens (Greenwald and Horvitz 1980). We made frozen glycerol stocks of these mutants and confirmed their recovery after approximately 1 month in cryogenic storage for all the mutants. For future genetic studies, the low male ratio after cross-mating will be an obstacle. Successive cross-mating using a limited number of males produced by each cross is possible with most of our mutants, but it is more laborious than with *C. elegans*. The isolation and use of Him (high incidence of males) mutants for mating might help overcome this problem. Furthermore, by using whole-genome sequencing techniques, we might achieve easier mapping and identification of mutations without any complex and time-consuming genetic mapping (Zuryn *et al.* 2010). Therefore, because of the ease of isolation of recessive mutants and making frozen stocks, *B. okinawaensis* has great potential for future biological studies.

### Mating preferences

*B. okinawaensis* males successfully mated with sperm-depleted old hermaphrodites (Table 2) and were chemotactically attracted only by sperm-depleted old hermaphrodites. A similar mating preference was also reported in *C. elegans* by Morsci *et al.* (2011), who found that *C. elegans* males preferentially mated with older hermaphrodites, dependent on the sperm status in the hermaphrodite. However, if *C. elegans* males are in physical proximity, then they display response behavior and can mate with young hermaphrodites (Barr and Garcia 2006). In *C. elegans*, male sexual behaviors are driven by long- and short-range chemical and contact-based physical signals from hermaphrodites (Barr and Garcia 2006). Therefore, *C. elegans* males would be stimulated by short-range chemical and/or physical mating signals from hermaphrodites possessing sperm. However, in *B. okinawaensis*, even if males had physical contact with young hermaphrodites still possessing their own self-produced sperm, males still did not display mating behavior, including response behavior, vulva location, and spicule insertion. This result suggests that young hermaphrodites possessing self-produced sperm lack the full repertoire of signals to attract a male.

### Sperm size

Our sperm size measurement revealed that there was no significant difference in the cross-sectional area of spermatids between males (12.2 μm^2^) and hermaphrodites (11.9 μm^2^). Lamunyon and Ward (1999) investigated the spermatid size of seven different hermaphrodite species of rhabditid nematodes, including *C. elegans*, and reported that hermaphrodites of the species examined produced smaller sperm than males. According to Baldi *et al.* (2011), the average cross-sectional areas of spermatids of males and hermaphrodites in the *C. elegans* N2 strain were 24.0 μm^2^ and 13.5 μm^2^, respectively. We also measured the cross-sectional area of male spermatids of the gonochoristic species *Bursaphelenchus xylophilus* (data not shown in Figure 7) and found the average cross-sectional area of male sperm in *B. xylophilus* to be 45.4 μm^2^. The spermatid size of *B. okinawaensis*, especially spermatids of males, is thus small compared with those of other nematodes. Lamunyon and Ward (1998) reported that larger sperm outcompete smaller sperm in *C. elegans*. If this finding is generalized to all nematodes, then we would not expect *B. okinawaensis* male sperm to outcompete hermaphrodite sperm. However, this would not be a disadvantage for males in sperm competition because male mating behavior is induced only by sperm-depleted hermaphrodites in *B. okinawaensis*. Because most *Bursaphelenchus* nematodes are gonochoristic, hermaphroditism in *B. okinawaensis* most likely has evolved from a gonochoristic species. In gonochoristic species, male–male sperm competition might be important because the male ratio in gonochoristic populations is generally approximately 50%. This severe competition could be a powerful force driving the evolution of sperm size and could explain why male sperm in gonochoristic species are much larger than those of males in hermaphroditic species (Lamunyon and Ward 1999). However, making smaller sperm would benefit the male by reducing energy costs. Baldi *et al.* (2011) argued that there is a developmental bias among the *Caenorhabditis* based on sexual phenotype; gonochoristic females that are genetically induced to become hermaphrodites nonetheless make smaller sperm, but both are larger than in hermaphroditic species. The equal size of male and hermaphrodite sperm in *B. okinawaensis* might result from the balance of multiple selective forces emanating from a harsh environment: energy conservation leading to a decrease in sperm size and a biased sex ratio and mating system (see below) that does not favor sperm size differences.

### Sex ratio distortion

Our mating experiments also gave a striking result: the sex ratio of cross-fertilized progeny in *B. okinawaensis* was highly skewed toward hermaphrodites (Table 2). For chromosomal sex determination systems, cross-mating should yield an approximately 1:1 ratio of males to hermaphrodites. However, there are many exceptions in nature (Gershenson 1928; Lyttle 1991). Sex ratio distortions after cross-mating are caused by multiple types of nuclear and cytoplasmic factors (Lyttle 1991). One of the well-known nuclear sex distortion mechanisms is meiotic drive, which causes preferential production of certain gametes during meiosis (Jaenike 1996; Sweeny and Barr 1978; Cazemajor *et al.* 2000; Jaenike 2001; James and Jaenike 1990). Supernumerary chromosomes such as the B chromosome appear to act as selfish genetic elements and distort the sex ratio (Werren 1991; Vicente *et al.* 1996; Camacho *et al.* 2011; Akbari *et al.* 2013). Cytoplasmic mechanisms of sex ratio distortion include the presence of microorganisms such as *Wolbachia*, which can induce distortion (Werren *et al.* 2008) via feminization (Bouchon *et al.* 1998), male-killing (Hurst 1991), and cytoplasmic incompatibility (Reed and Werren 1995). The sex ratio of many nematodes, especially parasitic species, is skewed toward females even if their reproductive mode is gonochoristic (Dix *et al.* 1994; Harvey *et al.* 2000; Yoshida *et al.* 2009; Shakes *et al.* 2011). However, there is no report that there is a highly biased sex ratio in *Bursaphelenchus* nematodes after cross-mating.

### Driving force of sex ratio distortion in *B. okinawaensis*

The sex distortion of *B. okinawaensis* might be due to their severe habitat, which is likely limited for food and space and has an abundance of antagonistic organisms. With the exception of several serious plant pathogen species like *B. xylophilus* and *B. cocophilus*, most *Bursaphelenchus* nematodes are free-living fungal feeding nematodes. They usually live in dead wood and propagate by feeding on fungi. The ecology of *B. okinawaensis* is still unclear, but Kanzaki *et al.* (2008) suggested that their habitat would be twigs of dead broad-leaved trees. During our strain collection on Ishigaki Island in June 2012, we isolated *B. okinawaensis* only from longhorn beetles, *M. maruokai*, which are endemic to Ishigaki and Iriomote Islands in Japan. The host plant of *M. maruokai* is some kind of broad-leaved tree. The climate of Ishigaki is subtropical, with an average temperature of 28°and 80% humidity in June. Furthermore, May and June comprise the rainy season on Ishigaki Island. During this season, *B. okinawaensis* could enter the newly dead or dying trees through holes in the bark that the vector beetle cut when laying its eggs. Therefore, wood decay would progress rapidly and the habitat of *B. okinawaensis* would become harsh immediately after invasion. Under this condition, we speculate that a 1:1 sex ratio would be at a selective disadvantage due to slow growth. Hermaphroditism and a hermaphrodite-biased sex ratio likely are advantageous for rapid reproduction, and thus could be selected on a short time scale. However, outcrossing increases the phenotypic space accessible to the population by allowing new combinations of alleles, and allows them to be selected for on a longer time scale. The balance of these forces could, in principle, lead to the particular sex ratio observed in the few populations sampled. Further genetic and ecological information is required to understand the forces that led to sex ratio distortion in *B. okinawaensis*.

A severe habitat would also influence their dauer formation mechanism. For dauer formation, species closely related to *B. okinawaensis* require a cue derived from the vector insect as well as unfavorable conditions (Warren and Linit 1993). However, *B. okinawaensis* can easily go into the dauer stage under unfavorable conditions (*e.g.*, food shortage, population density) without an insect-derived cue. This dauer formation feature would be an evolutionary adaptation to survive in such a severe environment.

## Conclusion

*Bursaphelenchus okinawaensis* has two sexes: a self-fertilizing hermaphrodite and a male. The generation time of this nematode is short (3.5 d and 3 d at 25° and 30°, respectively), and we can easily cultivate it on yeast plates by self- or cross-fertilization. Furthermore, *B. okinawaensis* has a unique life history (*i.e.*, fungal feeding, insect and plant association) and is phylogenetically distant from *Caenorabditis* and *Pristionchus*. Genome sequencing of *B. okinawaensis* is ongoing (R. Shinya, T. Kikuchi, I. Antoshechkin, and P. Sternberg, unpublished observations). Although the development of transgenic and knockout/knockdown techniques is needed for examining gene function effectively, the emergence of this new hermaphroditic *B. okinawaensis* will be useful for future studies about evolution of sexual allocation and determination systems, feeding behavior, and parasitism.
